# Novel Heme Oxygenase-1 (HO-1) Inducers Based on Dimethyl Fumarate Structure

**DOI:** 10.3390/ijms21249541

**Published:** 2020-12-15

**Authors:** Valeria Sorrenti, Luca Vanella, Chiara Bianca Maria Platania, Khaled Greish, Claudio Bucolo, Valeria Pittalà, Loredana Salerno

**Affiliations:** 1 Department of Drug Sciences, University of Catania, V.le A. Doria 6, 95125 Catania, Italy; sorrenti@unict.it (V.S.); lvanella@unict.it (L.V.); l.salerno@unict.it (L.S.); 2 Department of Biomedical and Biotechnological Sciences, Section of Pharmacology, School of Medicine, University of Catania, Via Santa Sofia 97, 95123 Catania, Italy; chiara.platania@unict.it (C.B.M.P.); claudio.bucolo@unict.it (C.B.); 3 Department of Molecular Medicine, College of Medicine and Medical Sciences, Princess Al-Jawhara Centre for Molecular Medicine, Arabian Gulf University, Manama 329, Bahrain; khaledfg@agu.edu.bh

**Keywords:** heme oxygenase (HO), HO-1 inducers, dimethyl fumarate (DMF), LX-2 cells, hepatic fibrosis

## Abstract

Novel heme oxygenase-1 (HO-1) inducers based on dimethyl fumarate (DMF) structure are reported in this paper. These compounds are obtained by modification of the DMF backbone. Particularly, maintaining the α, β-unsaturated dicarbonyl function as the central chain crucial for HO-1 induction, different substituted or unsubstituted phenyl rings are introduced by means of an ester or amide linkage. Symmetric and asymmetric derivatives are synthesized. All compounds are tested on a human hepatic stellate cell line LX-2 to assay their capacity for modifying HO-1 expression. Compounds **1b**, **1l** and **1m** stand out for their potency as HO-1 inducers, being 2–3 fold more active than DMF, and for their ability to reverse reactive oxygen species (ROS) production mediated using palmitic acid (PA). These properties, coupled with a low toxicity toward LX-2 cell lines, make these compounds potentially useful for treatment of diseases in which HO-1 overexpression may counteract inflammation, such as hepatic fibrosis. Docking studies show a correlation between predicted binding free energy and experimental HO-1 expression data. These preliminary results may support the development of new approaches in the management of liver fibrosis.

## 1. Introduction

Heme oxygenase (HO) is a microsomal enzyme which degrades heme in carbon monoxide (CO), free iron (Fe^2+^), biliverdin (BV) and bilirubin (BL) [1]. To date, three isoforms have been described in mammals (HO-1, HO-2, and HO-3). Considering these three isoforms, HO-1 is the most studied and it is strongly inducible in response to a variety of stimuli. These stimuli include reactive oxygen species (ROS), heat, hypoxia, its substrate heme, heavy metals, and cytokines [2]. Although it has been clearly demonstrated that an increase in HO-1 catalytic activity and/or expression is associated with specific serious pathologies, generally, HO-1 over-function is responsible for cytoprotection [3,4,5]. These last are obtained by antioxidant, anti-inflammatory, anti-proliferative, and anti-apoptotic properties ascribed directly or indirectly to the enzyme. Due to its multiple biological roles, pharmacologic modulation of the HO-1 system may represent an effective and synergistic strategy to ameliorate several diseases [6]. Particularly, controlled upregulation of this protein may be helpful in all those pathologies with oxidative and/or inflammatory components (atherosclerosis, carcinogenesis, ischemia-reperfusion or degenerative diseases, inflammatory processes) [7,8,9,10,11,12]. HO-1 expression is physiologically mostly under the control of the nuclear factor (erythroid-derived 2)-like 2 (Nrf2). Occurring at basal conditions, Nrf2 is located in the cytoplasm, linked to the Kelch-like ECH-associated repressor protein 1 (Keap1) to form the Keap1/Nrf2 inactive complex. After stressful or inflammatory stimuli, Nrf2 is released from the Keap1/Nrf2 complex, migrates into the nucleus, and initiates transcriptions of cytoprotective genes, including those for HO-1 production [13,14,15,16]. Nrf2/HO1 activation in response to oxidative stress has been demonstrated as beneficial in a number of diseases. Some examples include protection against lung injury in preterm newborns and lung fibrosis in adults, prevention of cardiovascular diseases associated with aging and vascular oxidative stress, mitigation of chronic pain and its associated comorbidities, and more [16,17,18,19].

Many natural, synthetic, and semi-synthetic molecules are described in literature as HO-1 inducers and their beneficial potential is widely recognized [20,21,22,23,24]. Seen in many cases, their function depends on interaction with the numerous cysteine residues present in the Keap1/Nrf2 complex. Indeed, although HO-1 inducers are chemically heterogeneous, most of them possess reactive electrophilic groups able to bind Keap1 cysteine residues, removing their repression role on Nrf2. They include resveratrol, quercetin, curcumin, caffeic acid phenethyl ester, and other polyphenols among natural compounds [25,26,27], and synthetic compounds such as dimethyl fumarate (DMF). DMF is an FDA (Food and Drug Administration) approved drug used for the treatment of relapsing-remitting multiple sclerosis (MS) and psoriasis. Its modulatory role of the Nrf2 cytoprotective pathway is well documented and is associated with the expression of a wide plethora of genes, including those encoding HO-1 [28,29,30,31].

Lately, our research group has been involved in numerous projects regarding the development of novel HO-1 modulators [32,33,34,35,36,37,38]. Regarding HO-1 induction, we recently described DMF derivatives and their properties as lung antifibrotic agents [39]. Continuing chemical modification of the parent DMF, in this study we design and synthesize a new series of DMF derivatives with the aim of obtaining more potent HO-1 inducers. We test all compounds in the human hepatic stellate cell (HSC)s line LX-2 to assay their capacity to modify HO-1 expression. Proliferation of HSCs plays a pivotal role in the progression of hepatic fibrosis consequent to chronic liver injury [40]. Several studies have suggested that Nrf2 and its downstream antioxidant factor HO-1 may contribute to improvement of liver fibrosis [41,42,43]. Anti-inflammatory effects obtained by an increase of HO-1 expression stimulated by novel synthetic compounds may be viewed as a new strategy able to counteract hepatic fibrosis. We also test LX-2 viability in the presence of all compounds using an MTT test to verify potential cytotoxicity and the ability of the most interesting derivatives to prevent ROS production. Finally, we perform a covalent docking approach to explore the covalent binding of these DMF derivatives to Cys151 of Keap1, which destabilization, in turn, promotes Nrf2 translocation, finally promoting HO-1 expression [39]. Molecular Mechanics Generalized Surface Area (MM-GBSA) calculation explains the differences in the experimentally observed activities.

## 2. Results and Discussions

### 2.1. Chemistry

**Scheme 1:**
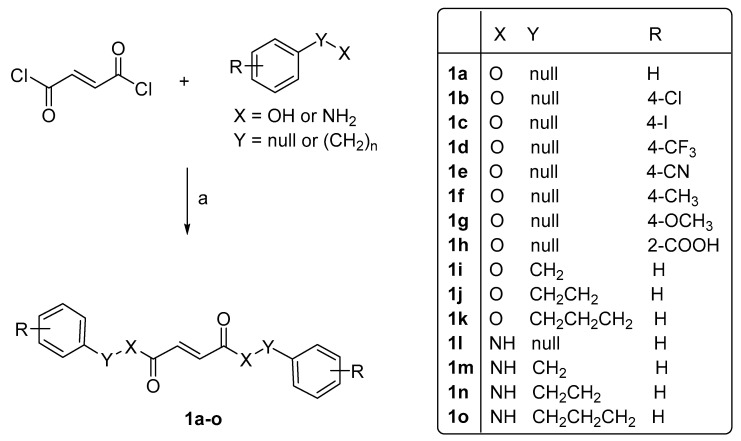
Reagents and conditions: (a) TEA, cyclohexane, or dichloromethane, 0 °C, 15 min, then 22 °C, 1 h.

**Scheme 2:**
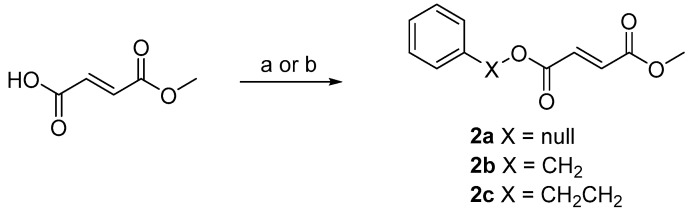
Reagents and conditions: (a) SOCl_2_, dry THF, 90 °C, 4 h, then phenol, TEA, 0 °C, 15 min, then 22 °C, 6 h; (b) (bromomethyl) or (bromoethyl)benzene, K_2_CO_3_, KI, acetone, 40 °C, 16 h.

The synthesis of derivatives **1a**–**o** and **2a**–**c** was achieved as reported in Scheme 1 and Scheme 2. Briefly, compounds **1a**–**o** were synthesized in good yields using the corresponding alcohol, phenol, or amine derivatives as a starting material and treating them with fumaryl chloride dissolved in dry cyclohexane or dichloromethane, at room temperature (Scheme 1).

The synthesis of **2a** was accomplished by transforming (*E*)-4-methoxy-4-oxobut-2-enoic acid in the correspondent acyl chloride upon treatment with thionyl chloride (SOCl_2_) and then adding phenol in the presence of TEA and dry THF as a solvent to the obtained mixture (Scheme 2). Compounds **2b**,**c** were obtained by treating (*E*)-4-methoxy-4-oxobut-2-enoic acid with (bromomethyl) or (bromoethyl)benzene in the presence of potassium carbonate (K_2_CO_3_), potassium iodide (KI), and using acetone as the solvent (Scheme 2). The target products were characterized by IR, ^1^H-NMR, ^13^C-NMR spectra (Supporting Information, Figures S1–S8), and elemental analysis, and were consistent with the proposed structures.

### 2.2. Rational Design and Biological Evalution

During the twentieth century, scientists demonstrated a certain disbelief toward the design of drugs acting through covalent interactions at the target. Major concerns were related to scarce selectivity and probable idiosyncratic reactions [44]. Conversely, the latest FDA approval of drugs behaving as weak electrophilic, e.g., dimethyl fumarate (DMF), ibrutinib, neratinib, and afatinib, cleared the way for the development of a new generation of compounds capable of covalently binding cysteine at the target [45]. Among electrophilic drugs, DMF can be considered a convenient backbone for the design of compounds increasing heme oxygenase inducer (HO-1) expression levels due to its mild electrophile nature [46]. Compounds **1a**–**o** and **2a**–**c,** hence, were designed and synthesized (Table 1). Accompanying the aim of investigating the structure-activity relationships (SARs) of compounds **1a**–**o** and **2a**–**c** as HO-1 inducers, we measured the HO-1 expression by ELISA test in lysates of the human hepatic stellate cell (HSC) line LX-2. DMF was used as a positive control. Many of the new derivatives are endowed with HO-1 inducing activity, showing potency comparable or better than DMF, especially at the lower doses. Previously demonstrated [39], substituents at the 1– and 4–position of fumaric acid are essential factors for ligand binding, by establishing extra non-covalent interactions with the target. Based on this, we added phenyl rings, linked directly to the carboxylic moieties (compound **1a**) or through a short connecting chain (1–3 methylene units, compounds **1i**–**k**). The same substitution pattern was applied to asymmetrically functionalize one carboxylic acid residue of fumaric acid monomethyl ester (compounds **2a**–**c**). This last modification also was supported by the activity of monomethyl fumarate as an HO-I inducer [30,47]. Among these compounds, **1a** showed the most interesting levels of activity, comparable or better than DMF. Considering that compound **1a** displayed the highest activity as an HO-1 inducer with respect to asymmetric **2a**–**c** and homologues **1i**–**k**, in a second step we decorated the phenyl rings of **1a** with substituents possessing different electronegativity, obtaining compounds **1b**–**h**. Generally, electron-releasing groups gave the worst results (compounds **1f** and **1g**), whereas, with the exception of CF_3_, the presence of an electron-withdrawing group such as 4-Cl, 4-I and 2-COOH (compounds **1b**, **1c** and **1h**) increased the potency as an HO-1 inducer; particularly noteworthy was the 4-Cl derivate **1b**. Finally, with the aim of further study of the SARs of our compounds, the ester function was substituted with an amide residue (compounds **1l**–**o**). This substitution highly improved the potency, with **1l** and **1m** together with **1b** being the most potent compounds of the whole series. However, while compounds **1b** and **1l** showed a progressive increase in activity, compound **1m** showed a peak in activity at 5 μM (14.15 fold induction) and a slight reduction at 10 μM (13.76 fold induction). Cytotoxicity was tested using an MTT assay performed in the same cell line. Almost all compounds can be considered as safe as DMF in LX-2. However, it should be noticed that compounds **1c**, **1g**, **1i** and **1m** influenced, to a certain, extent the viability of LX-2 cells (IC_50_ values 20.8, 22.3, 17.7 and 18.6 μM, respectively).

**Figure 1:**
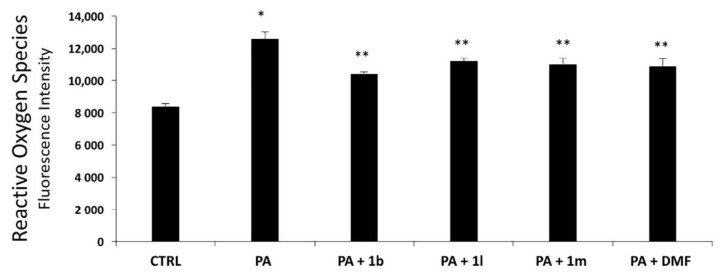
Determination of reactive oxygen species (ROS) using DCFH-DA in LX-2 cells in the presence of palmitic acid (PA), and PA in co-treatment with **1b**, **1l**, **1m** and dimethyl fumarate (DMF). * *p* < 0.05 vs. control; ** *p* < 0.05 vs. PA.

Concerning the most interesting compounds, **1b**, **1l** and **1m**, we tested their ability for reactive oxygen species (ROS) production mediated by palmitic acid (PA). The obtained results showed that PA increased ROS production; however, **1b**, **1l**, **1m** and DMF co-treatment significantly reversed the effect mediated by PA, as reported in Figure 1. The compounds alone did not significantly increase the release in ROS (data not shown).

Hepatic fibrosis is characterized by the excessive accumulation of an extracellular matrix, primarily collagen, within the liver. Previously mentioned, the nuclear factor (erythroid-derived 2)-like 2 (Nrf2) pathway, along with HO-1 overexpression, is associated with the induction of the most pivotal endogenous antioxidative system; its activation is able to suppress hepatic steatosis and mitigate liver fibrosis, principally through inhibition of oxidative stress and HO-1 mediated human hepatic stellate cell (HSCs) ferroptosis [48,49,50,51]. Therefore, on this basis, the newly identified potent HO-1 inducers seem to be promising for further studies as liver antifibrotic agents.

### 2.3. Computational Studies

**Figure 2:**
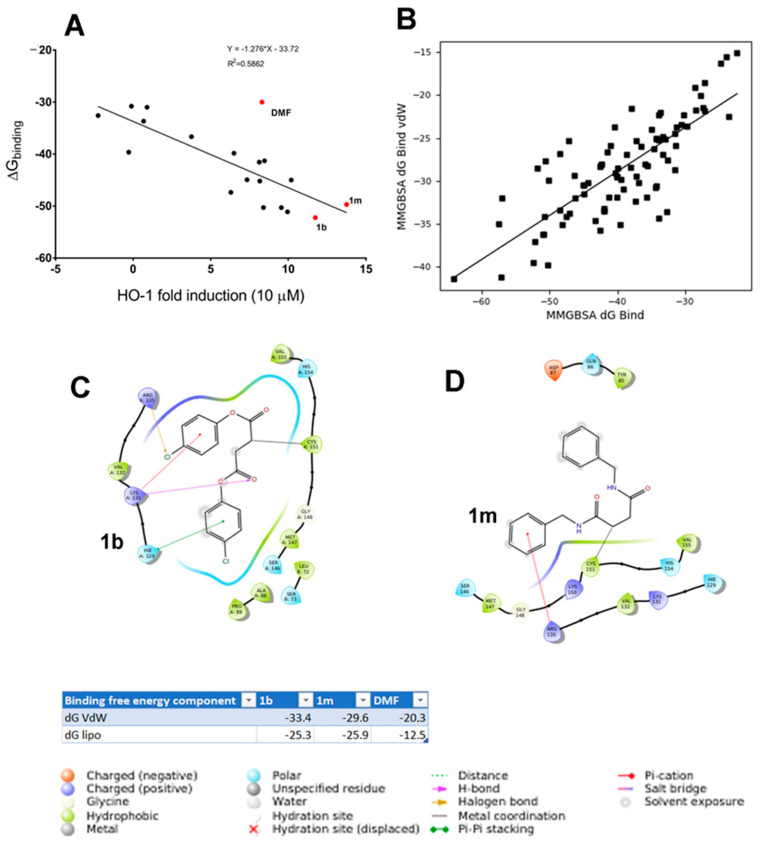
Results of computational studies. (**A**) Correlation of experimental results with predicted binding free energy; (**B**) correlation of predicted binding free energy versus Van der Waal component; (**C**) pose of compound **1b** at Kelch-like ECH-associated repressor protein 1 (Keap1); (**D**) pose of compound **1m** at Keap1.

To further explore the ability of these compounds to activate the upstream nuclear factor (erythroid-derived 2)-like 2 (Nrf2) pathway and, in turn, heme oxygenase inducer (HO-1) expression, we performed in silico studies. We carried out a covalent docking calculation of this new series of dimethyl fumarate (DMF) derivatives, predicting the covalent binding (Michael Addition) of compounds at Cys151 of the Kelch-like ECH-associated repressor protein 1 (Keap1), to correlate the HO-1 fold induction with predicted ΔG_binding_ (MM-GBSA rescoring). This computational approach has been previously studied [39]. Experimental HO-1-fold regulation correlated (R^2^ = 0.586) with predicted ΔG_binding_ (Figure 2A). This result strengthens the previously published data about the design of anti-oxidant compounds, based on covalent docking at a key Cys151 residue of Keap1 [39]. Destabilization of the Keap1 dimer led to Nrf2 translocation, then to promotion of expression of the anti-oxidant machinery proteins, such as HO-1. The predicted MM-GBSA ΔG_binding_ correlated with the binding free energy component related to the Van der Waal non-covalent interaction (Figure 2B).

Compared to DMF, the two hit HO-1 inducers **1b** (Figure 2C) and **1m** (Figure 2D) showed the best predicted binding free energy due to favorable Van der Waal and hydrophobic interactions (Figure 2 more negative ΔG_binding_, and components of predicted binding free energy components dG_VdW_ and dG_lipo_).

## 3. Materials and Methods

### 3.1. Chemistry

Reactions were followed by thin layer chromatography (TLC) performed on Sigma–Aldrich silica plates (60 F254), using UV light (254 nm and 366 nm) for visualization, and developed using I2 chamber. Merck silica gel 60, 0.040–0.063 mm (230–400 mesh) was used for flash chromatographic purification on glass columns. Melting points were assigned using an IA9200 Electrothermal apparatus furnished with a digital thermometer in capillary glass tubes and were uncorrected. Infrared spectra (IR) were recorded on a spectrometer in KBr disks or NaCl crystal windows. ^1^H and ^13^C NMR spectra were recorded on a 200 or 500 MHz spectrometer in DMSO-*d*_6_ solution. Chemical shifts were given in δ values (ppm), using tetramethylsilane (TMS) as the internal standard; coupling constants (*J*) were given in Hz. Signal multiplicities were characterized as s (singlet), d (doublet), t (triplet), q (quartet), m (multiplet), or br (broad). Elemental analyses for C, H, N, and O were within ±0.4% of theoretical values and were accomplished through a Carlo Erba Elemental Analyzer Mod. 1108 apparatus. Reagents, solvents, and starting materials were acquired from commercial suppliers.

### 3.2. General Procedure for the Synthesis of (2E)-2-Butenedioic Acid Derivatives *(**1a**–**k**)*

Fumaryl chloride (1.5 mmol) dissolved in 4 mL of dry cyclohexane was slowly added to an ice-cooled solution of the suitable alcohol (3 mmol) and TEA (3 mmol) in 16 mL of dry cyclohexane. The addition was completed in about 15 min, then the reaction slurry was stirred at 22 °C for 2 h. After this time, 200 mL of EtOAc was added to the mixture and the organic phase was washed with HCl 0.1 N (2 × 150 mL), a solution of Na_2_CO_3_ 5% (2 × 150 mL), and brine (1 × 150 mL). The organic layer was dried over anhydrous Na_2_SO_4_, filtered, and evaporated. The obtained crudes were purified by flash chromatography or recrystallization. The suitable purification process and chemical characterization of derivatives **1a**–**k** are given below.

#### 3.2.1. (2*E*)-2-Butenedioic acid, 1,4-diphenyl ester (**1a**)

The crude material was purified by flash chromatography using cyclohexane/ethyl acetate (6:4) as an eluant to present the title compound as a white solid (65%): mp 164–165 °C; IR (KBr) cm^−1^ 1735, 1588, 1307, 1260, 1158, 1149, 970, 761, 730; ^1^H NMR (200 MHz, DMSO-*d*_6_) δ 7.51–7.46 (m, 4H, aromatic), 7.35–7.31 (m, 2H, aromatic), 7.28–7.24 (m, 4H, aromatic), 7.19 (s, 2H, COCH=CHCO). Analysis calculated for C_16_H_12_O_4_: C, 71.64; H, 4.51, found: C, 71.84; H, 4.45.

#### 3.2.2. (2*E*)-2-Butenedioic acid, 1,4-bis(4-chlorophenyl) ester (**1b**)

The crude material was recrystallized from EtOH to present the title compound as a white solid (53%): mp 178–179 °C; IR (KBr) cm^−1^ 1742, 1488, 1294, 1200, 1137, 1091, 974, 720; ^1^H NMR (500 MHz, DMSO-*d*_6_) δ 7.56–7.52 (m, 4H, aromatic), 7.33–7.30 (m, 4H, aromatic), 7.19 (s, 2H, COCH=CHCO); ^13^C NMR (125 MHz, DMSO-d6): δ 162.75, 148.77, 133.81, 130.46, 129.55, 123.55. Analysis calculated for C_16_H_10_Cl_2_O_4_: C, 57.00; H, 2.99, found: C, 57.25; H, 3.01.

#### 3.2.3. (2*E*)-2-Butenedioic acid, 1,4-bis(4-iodophenyl) ester (**1c**)

The crude material was recrystallized from EtOH to present the title compound as a white solid (39%): mp 266–267 °C; IR (KBr) cm^−1^ 1732, 1477, 1304, 1196, 1143, 1007, 844, 783; ^1^H NMR (500 MHz, DMSO-*d*_6_) δ 7.83–7.80 (m, 4H, aromatic), 7.17 (s, 2H, COCH=CHCO), 7.12–7.08 (m, 4H, aromatic); ^13^C NMR (125 MHz, DMSO-*d_6_*) δ 162.67, 149.89, 138.38, 133.82, 124.13, 91.20. Analysis calculated for C_16_H_10_I_2_O_4_: C, 36.95; H, 1.94, found: C, 37.31; H, 1.91.

#### 3.2.4. (2*E*)-2-Butenedioic acid, 1,4-bis(4-trifluoromethyphenyl) ester (**1d**)

The crude material was purified by flash chromatography using cyclohexane/ethyl acetate (9:1) as an eluant to present the title compound as a white solid (44%): mp 122–123 °C; IR (KBr) cm^−1^ 1743, 1322, 1291, 1199, 1171, 1134, 1103, 1062, 873, 784; ^1^H NMR (200 MHz, DMSO-*d*_6_) δ 7.94–7.78 (m, 4H, aromatic), 7.62–7.40 (m, 4H, aromatic), 7.25 (s, 2H, COCH=CHCO). Anal. calcd. for C_18_H_10_F_6_O_4_: C, 53.48; H, 2.49, found: C, 53.31; H, 2.39.

#### 3.2.5. (2*E*)-2-Butenedioic acid, 1,4-bis(4-cyanophenyl) ester (**1e**)

The crude material was recrystallized from EtOH to present the title compound as a white solid (57%): mp 230–232 °C; IR (KBr) cm^−1^ 1735, 1504, 1212, 1172, 1189, 860, 744; ^1^H NMR (200 MHz, DMSO-*d*_6_) δ 8.04–7.95 (m, 4H, aromatic), 7.58–7.41 (m, 4H, aromatic), 7.24 (s, 2H, COCH=CHCO). Anal. calcd. for C_18_H_10_N_2_O_4_: C, 67.93; H, 3.17, found: C, 67.71; H, 3.21.

#### 3.2.6. (2*E*)-2-Butenedioic acid, 1,4-bis 4-methylphenyl) ester (**1f**)

The crude material was recrystallized from EtOH to present the title compound as a white solid (27%): mp 160–161 °C; IR (KBr) cm^−1^ 1739, 1504, 1288, 1188, 1162, 1137, 1102, 991, 811, 764; ^1^H NMR (200 MHz, DMSO-*d*_6_) δ 7.33–7.21 (m, 4H, aromatic), 7.19–7.11 (m, 4H, aromatic), 7.10 (s, 2H, COCH=CHCO), 2.33 (s, 6H, CH_3_). Anal. calcd. for C_18_H_16_O_4_: C, 72.96; H, 5.44, found: C, 73.04; H, 5.29.

#### 3.2.7. (2*E*)-2-Butenedioic acid, 1,4-bis(4-methoxyphenyl) ester (**1g**)

The crude material was recrystallized from EtOH to present the title compound as a white solid (59%): mp 180–181 °C; IR (KBr) cm^−1^ 1741, 1507, 1458, 1286, 1248, 1191, 1129, 1026, 953, 781; ^1^H NMR (200 MHz, DMSO-*d*_6_) δ 7.22–7.18 (m, 4H, aromatic), 7.05–6.99 (m, 4H, aromatic), 6.98 (s, 2H, COCH=CHCO), 3.77 (s, 6H, CH_3_O). Anal. calcd. for C_18_H_16_O_6_: C, 65.85; H, 4.91, found: C, 65.94; H, 4.99.

#### 3.2.8. (2*E*)-2-Butenedioic acid, 1,4-bis(2-carboxyphenyl) ester (**1h**)

The crude material was recrystallized from EtOH to present the title compound as a white solid (67%): mp 193–194 °C; IR (KBr) cm^−1^ 1741, 1697, 1303, 1203, 1136, 1084, 891, 763; ^1^H NMR (200 MHz, DMSO-*d*_6_) δ 8.02–7.97 (m, 2H, aromatic), 7.78–7.64 (m, 2H, aromatic), 7.52–7.29 (m, 4H, aromatic), 7.19 (s, 2H, COCH=CHCO). Anal. calcd. for C_18_H_12_O_8_: C, 60.68; H, 3.40, found: C, 60.49; H, 3.37.

#### 3.2.9. (2*E*)-2-Butenedioic acid, 1,4-bis(phenylmethyl) ester (**1i**)

The crude material was recrystallized from EtOH to present the title compound as a white solid (67%): mp 59–61 °C; IR (KBr) cm^−1^ 3032, 1708, 1457, 1380, 1292, 1149, 1005, 965, 754; ^1^H NMR (500 MHz, DMSO-*d*_6_) δ 7.43–7.32 (m, 10H, aromatic), 6.86 (s, 2H, COCH=CHCO), 5.23 (s, 4H, CH_2_O); ^13^C NMR (125 MHz, DMSO-*d*_6_) δ 164.11, 135.48, 133.27, 128.45, 128.21, 66.50. Anal. calcd. for C_18_H_16_O_4_: C, 72.96; H, 5.44, found: C, 72.74; H, 5.45.

#### 3.2.10. (2*E*)-2-Butenedioic acid, 1,4-bis(phenylethyl) ester (**1j**)

The crude material was recrystallized from EtOH to present the title compound as an off white solid (60%): mp 77–79 °C; IR (KBr) cm^−1^ 3080, 2941, 1715, 1638, 1499, 1474, 1253, 1162, 982, 755; ^1^H NMR (500 MHz, DMSO-*d*_6_) δ 7.35–7.17 (m, 10H, aromatic), 6.67 (s, 2H, COCH=CHCO), 4.36 (t, 4H, *J* = 6.8 Hz, OCH_2_CH_2_), 2.94 (t, 4H, *J* = 6.8 Hz, OCH_2_CH_2_); ^13^C NMR (125 MHz, DMSO-*d*_6_) 164.08, 137.70, 133.07, 128.81, 128.33, 126.40, 65.44, 34.08. Anal. calcd. for C_20_H_20_O_4_: C, 74.06; H, 6.22. Found: 74.33; H 6.20.

#### 3.2.11. (2*E*)-2-Butenedioic acid, 1,4-bis(phenylpropyl) ester (**1k**)

The obtained residue was purified using a Biotage^®^ chromatographic system with Biotage^®^ SNAP KP-Sil flash chromatography cartridges using cyclohexane/ethyl acetate (9.5:0.5) as an eluant to present the title compound as a sticky colorless oil (65%): IR (KBr) cm^−1^ 1738, 1590, 1317, 1265, 1160, 1152, 980, 769, 730; ^1^H NMR (200 MHz, DMSO-*d*_6_) δ 7.34–7.13 (m, 10H, aromatic), 6.74 (s, 2H, COCH=CHCO), 4.16 (t, 4H, *J* = 6.6 Hz, OCH_2_), 2.68 (t, 4H, *J* = 7.8 Hz, PhCH_2_), 1.95 (m, 4H, CH_2_C*H*_2_CH_2_). Anal. calcd. for C_22_H_24_O_4_: C, 74.98; H, 6.86, found: C, 74.84; H, 6.75.

### 3.3. General Procedure for the Synthesis of (E)-N, N’-Diphenylalkyl-Butenediamide Derivatives *(**1l**–**o**)*

Fumaryl chloride (2 mmol) dissolved in 2 mL of dry dichloromethane was slowly added to an ice-cooled suspension of the suitable amine (4 mmol) and TEA (4 mmol) in 4 mL of dry dichloromethane. The addition was completed in about 15 min, then the reaction slurry was stirred at 22 °C, for 1 h. After this time, the precipitate was filtered under a vacuum and triturated with HCl 0.5 N (10 mL), NaHCO_3_ 5% (10 mL) and deionized water (10 mL). The obtained solids were recrystallized from ethanol. Suitable chemical characterization of derivatives **1l**–**o** is given below.

#### 3.3.1. (*E*)-*N*, *N*’-Diphenyl-2-butenediamide (**1l**)

Whitish solid (26%): mp 332–333 °C; IR (KBr) cm^−1^ 3305, 1652, 1598, 1540, 1443, 1339, 1159, 960, 757, 745; ^1^H NMR (200 MHz, DMSO-*d*_6_) δ 10.56 (s, 2H, NH), 7.71 (d, 4H, *J* = 7.6 Hz, aromatic), 7.36 (t, *J* = 7.4 Hz, 4H, aromatic), 7.21 (s, 2H, COCH=CHCO), 7.11 (t, *J* = 7.6 Hz, 2H, aromatic). Anal. calcd. for C_16_H_14_N_2_O_2_: C, 74.98; H, 6.86; N, 10.52, found: C, 74.88; H, 6.84; N, 10.53.

#### 3.3.2. (*E*)-*N*, *N*’-Diphenylmethyl-2-butenediamide (**1m**)

White solid (65%): mp dec.; IR (KBr) cm^−1^ 3282, 3085, 1630, 1561, 1493, 1454, 1433, 1338, 1242, 1191, 1081, 1035, 988, 695; ^1^H NMR (500 MHz, DMSO-*d*_6_) δ 8.91 (t, *J* = 5.0 Hz, 2H, NH), 7.33 (t, *J* = 7.5 Hz, 4H, aromatic), 7.27–7.23 (m, 6H, aromatic), 6.93 (s, 2H, COCH=CHCO), 4.38 (d, *J* = 6.0 Hz, 4H, PhCH_2_); ^13^C NMR (125 MHz, DMSO-*d*_6_) 163.69, 138.92, 132.77, 128.33, 127.28, 126.89, 42.32. Anal. calcd. for C_18_H_18_N_2_O_2_: C, 73.45; H, 6.16; N, 9.52, found: C, 73.38; H, 6.15; N, 9.54.

#### 3.3.3. (*E*)-*N*, *N*’-Diphenylethyl-2-butenediamide (**1n**)

White solid (60%): mp 274-275 °C; IR (KBr) cm^−1^ 3290, 1618, 1551, 1343, 1188, 994, 736, 700; ^1^H NMR (500 MHz, DMSO-*d*_6_) δ 8.47 (t, *J* = 5.5 Hz, 2H, NH), 7.31–7.28 (m, 4H, aromatic), 7.22–7.19 (m, 6H, aromatic), 6.79 (s, 2H, COCH=CHCO), 3.38 (m, 4H, NHC*H_2_*), 2.75 (t, *J* = 7.5 Hz, 4H, PhC*H_2_*); ^13^C NMR (125 MHz, DMSO-*d*_6_) δ 163.65, 139.25, 132.52, 128.59, 128.29, 126.10, 40.36, 34.84. Anal. calcd. for C_20_H_22_N_2_O_2_: C, 74.51; H, 6.88; N, 8.69, found: C, 74.33; H, 6.87; N, 8.71.

#### 3.3.4. (*E*)-*N*, *N*’-Diphenylpropyl-2-butenediamide (**1o**)

White solid (69%): mp 233–235 °C; IR (KBr) cm^−1^ 3285, 1628, 1564, 1451, 1340, 1200, 985, 715, 696; ^1^H NMR (200 MHz, DMSO-*d*_6_) δ 8.44 (s, 2H, NH), 7.32–7.19 (m, 10H, aromatic), 8.84 (s, 2H, COCH=CHCO), 3.18-3.14 (m, 4H, NHC*H_2_*), 2.60 (t, *J* = 7.6 Hz, 4H, PhC*H_2_*), 1.73 (m, 4H, CH_2_C*H*_2_CH_2_). Anal. calcd. for C_22_H_26_N_2_O_2_: C, 75.40; H, 7.48; N, 7.99, found: C, 75.28; H, 7.47; N, 8.00.

### 3.4. Synthesis of (2*E*)-2-Butenedioic Acid, Methyl Phenyl Ester *(**2a**)*

(2*E*)-2-Butenedioic acid 1-methyl ester (2 mmol) was dissolved in 4 mL of dry THF and 2 mmol of SOCl_2_ was slowly added. The reaction was heated to 90 °C under magnetic stirring for 4 h. After this time, the mixture was ice-cooled to 0 °C and 2 mmol of phenol and 2 mmol of TEA was added. The obtained suspension was stirred for 6 h at room temperature, then diluted with 100 mL of EtOAc and washed with HCl 0.1 N (2 × 75 mL), a solution of Na_2_CO_3_ 5% (2 × 75 mL), and brine (1 × 750 mL). The organic layer was dried over anhydrous Na_2_SO_4_, filtered, and evaporated. The crude was purified by flash chromatography using cyclohexane/ethyl acetate (9:1) as an eluant to present the title compound as a white solid (65%): mp 54–55 °C; IR (KBr) cm^−1^ 1736, 1718, 1311, 1259, 1178, 1161, 952, 755; ^1^H NMR (200 MHz, DMSO-*d*_6_) δ 7.50–7.20 (m, 5H, aromatic), 6.99 (s, 2H, COCH=CHCO), 3.79 (s, 3H, CH_3_). Anal. calcd. for C_11_H_10_O_4_: C, 64.08; H, 4.89, found: C, 64.17; H, 4.84.

### 3.5. General Procedure for the Synthesis of (2E)-2-Butenedioic Acid Derivatives ***2b*** and ***2c***

(2*E*)-2-Butenedioic acid 1-methyl ester (1 mmol) was dissolved in 10 mL of acetone and 3 mmol of the (bromomethyl) or (bromoethyl)benzene (for the synthesis of **2b** or **2c**, respectively), 1.2 mmol of K_2_CO_3_, and a catalytic amount of KI were added. The reaction was heated to 40 °C under magnetic stirring for 16 h. After this time, the mixture was filtered and concentrated under reduced pressure. The crude was purified by flash chromatography using cyclohexane/ethyl acetate (9:1) as an eluant to provide the desired final compounds **2b** and **2c**.

#### 3.5.1. (2*E*)-2-Butenedioic acid, methyl phenylmethyl ester (**2b**)

The title compound presented as a white solid (65%): mp 50–51 °C; IR (KBr) cm^−1^ 1718, 1301, 1215, 1156, 981, 755, 700; ^1^H NMR (200 MHz, DMSO-*d*_6_) δ 7.45–7.37 (m, 5H, aromatic), 6.83 (s, 2H, COCH=CHCO), 5.24 (s, 2H, CH_2_), 3.75 (s, 3H, CH_3_). Anal. calcd. for C_12_H_12_O_4_: C, 65.45; H, 5.49, found: C, 65.29; H, 5.40.

#### 3.5.2. (2*E*)-2-Butenedioic acid, methyl phenylethyl ester (**2c**)

The title compound presented as a white solid (25%): mp 44–45 °C; IR (KBr) cm^−1^ 1722, 1303, 1154, 1007, 983, 756; ^1^H NMR (200 MHz, DMSO-*d*_6_) δ 7.38–7.18 (m, 5H, aromatic), 6.73 (s, 2H, COCH=CHCO), 4.37 (s, *J* = 6.8 Hz, 2H, OCH_2_), 3.74 (s, 3H, CH_3_), 2.95 (t, *J* = 6.8 Hz, 2H, CH_2_Ph). Anal. calcd. for C_13_H_14_O_4_: C, 64.66; H, 6.02, found: C, 66.87; H, 6.11.

### 3.6. Biology

#### 3.6.1. Cell Culture of LX2 Cells

LX 2 cell lines were bought from American Type Culture Collection (Rockville, MD, USA). Upon defrosting, the cells were seeded in 6-well plates and maintained in a medium DMEM low glucose supplemented with 10% FBS and a 1% Penicillin and Streptomycin solution. The obtained cell cultures were left at 37 °C in an incubator with 5% CO_2_, and the culture medium was changed after 3 or 4 days. Sub-confluent cells were treated over a period of 24 h using various concentrations (1, 5, 10 µM) of compounds **1a**–**o**, **2a**–**c**, and dimethyl fumarate (DMF) previously dissolved in DMSO. Control groups were treated with only DMSO.

#### 3.6.2. ELISA Heme Oxygenase Inducer (HO-1) Measurements

The commercially available ELISA kit (ADI-EKS-800, Enzo Life Sci) was used to measure heme oxygenase inducer (HO-1) protein concentration in LX2 cellular lysates. The test was performed in agreement with the protocol reported by the manufacturer. Absorbance at λ = 450 nm was measured and the concentration of HO-1 was calculated from a standard curve previously created using purified HO-1 protein. Results were expressed as folds of change compared to the control group. Individual measurement was performed in triplicate and data were reported as averages.

#### 3.6.3. MTT Assay

An MTT assay was performed to monitor cell viability, measuring the conversion of tetrazolium salt to yield colored formazan, the amount of which was proportional to the number of living cells. Regarding this assay, LX2 cells were seeded at a concentration of 2 × 10^5^ cells per well, in a 96-multiwell flat-bottomed 200 μL microplate. The optical density of each well was measured with a microplate spectrophotometer reader (Synergy HT, BioTek) at λ = 570 nm.

#### 3.6.4. Reactive Oxygen Species (ROS) Assay

Determination of the reactive oxygen species (ROS) was performed using a fluorescent probe 2′,7′-dichlorofluorescein diacetate (DCFH-DA). The fluorescence (corresponding to the oxidized radical species 2′,7′-dichlorofluorescein, DCF) was monitored spectrofluorometrically (excitation, λ = 488 nm; emission, λ = 525 nm). Results were reported as fluorescence intensity. Concerning the experiments, the cells were seeded in 96-well plates at a density of 1 × 10^4^ cells per well. Then, the cells were treated for 24 h with DMEM containing a no cytotoxic concentration of palmitic acid (PA) 300 μM in the presence or absence of compound **1b**, **1l**, **1m** and dimethyl fumarate (DMF) (5 μM).

### 3.7. Computational Study

The computational approach hereby used, paralleled the same methods (covalent docking Michael Addition, MM-GBSA calculation) already carried out in our previous paper [39], using a different compound dataset (Table 1). Predictions were carried out within the Schrodinger Maestro^®^ suite. Plots were built by means of GraphPad Prism.

## 4. Conclusions

The present study accounts for the identification of dimethyl fumarate (DMF) analogs as heme oxygenase (HO-1) inducers. Modification of the DMF skeleton by adding phenyl groups differently adorned and spaced, and/or an amide function, was an effective approach to the design of potent HO-1 inducers. Derivatives **1b**, **1l** and **1m**, devoid of cytotoxic activity, were able to induce HO-1 expression at high levels and more effectively than the parent DMF. Compound **1m** showed a marked increase in HO-1 activity (a more than 7 and 14 times fold induction at 1 and 5 μM, respectively), in LX-2 cells. Additionally, compounds **1b**, **1l**, **1m**, and DMF reduced the reactive oxygen species (ROS) production induced by palmitic acid (PA) treatment. Demonstrated by in silico studies, and in accordance with earlier studies, these compounds seemed to be able to activate nuclear factor (erythroid-derived 2)-like 2 (Nrf2) and, in turn, HO-1 expression. To our knowledge, this is the first time DMF analogs have been reported as potent inducers of HO-1 expression in the liver cell line. These preliminary results may support the development of new approaches in the management of liver fibrosis. Further in vivo studies are necessary to confirm this hypothesis.

## Figures and Tables

**Table 1:** heme oxygenase inducers (HO-1) amount determined by ELISA for compounds **1a**–**o** and **2a**–**c** in LX-2 cells lysate.

Compound	Fold Induction ^1^ (ELISA)			MTT IC_50_ (μM)
	1 μM	5 μM	10 μM	
**1a**	6.65 ± 0.15	7.93 ± 0.18	8.16 ± 0.32	>50
**1b**	8.81 ± 0.20	8.19 ± 0.21	11.74 ± 0.41	>50
**1c**	6.02 ± 0.13	7.70 ± 0.19	8.13 ± 0.23	20.8 ± 1.5
**1d**	(−0.95) ± 0.02	1.40 ± 0.03	0.68 ± 0.02	>50
**1e**	3.68 ± 0.21	5.22 ± 0.11	6.50 ± 0.17	>50
**1f**	1.82 ± 0.09	3.94 ± 0.13	3.76 ± 0.15	>50
**1g**	0.30 ± 0.01	0.76 ± 0.02	0.90 ± 0.06	22.3 ± 1.8
**1h**	6.37 ± 0.22	7.45 ± 0.21	9.95 ± 0.19	>50
**1i**	3.40 ± 0.18	5.86 ± 0.19	7.34 ± 0.28	17.7 ± 1.3
**1j**	2.29 ± 0.10	5.91 ± 0.17	8.47 ± 0.33	>50
**1k**	2.26 ± 0.09	5.98 ± 0.16	9.55 ± 0.27	>50
**1l**	6.90 ± 0.14	9.74 ± 0.25	10.19 ± 0.29	>50
**1m**	7.46 ± 0.16	14.15 ± 0.34	13.76 ± 0.35	18.6 ± 1.4
**1n**	5.82 ± 0.12	7.52 ± 0.21	8.40 ± 0.38	>50
**1o**	4.85 ± 0.13	6.93 ± 0.23	6.30 ± 0.21	>50
**2a**	(−2.26) ± 0.08	(−2.15) ± 0.04	(−2.25) ± 0.02	>50
**2b**	(−1.61) ± 0.06	(−0007) ± 0.001	(0.28) ± 0.01	>50
**2c**	(−0.88) ± 0.02	(−0.60) ± 0.01	(−0.11) ± 0.01	>50
DMF	2.78 ± 0.09	7.63 ± 0.31	8.3 ± 0.23	>50

^1^ Data are shown as fold induction at values in μM ± standard deviation (SD). Values are the mean of triplicate experiments.

## Supplementary Materials

Supplementary materials can be found at https://www.mdpi.com/1422-0067/21/24/9541/s1.
