# Supplementary material for: Novel Heme Oxygenase-1 (HO-1) Inducers Based on Dimethyl Fumarate Structure
Source: Int J Mol Sci. 2020 Dec 15;21(24):9541. doi: 10.3390/ijms21249541 (PMC7765375; doi:10.3390/ijms21249541)
Supplement: Supplementary file 1 [file ijms-21-09541-s001.pdf]

International Journal of *Molecular Sciences*

MDPI

Communication

Novel Heme Oxygenase-1 (HO-1) Inducers Based on Dimethyl Fumarate Structure

Valeria Sorrenti [1], Luca Vanella [1], Chiara Bianca Maria Platania [2], Khaled Greish [3], Claudio Bucolo [2], Valeria Pittalà [1,*], Loredana Salerno [1]

[1] Department of Drug Sciences, University of Catania, V.le A. Doria 6, 95125 Catania, Italy; vpittala@unict.it (V.P.); sorrenti@unict.it (V.S.); lvanella@unict.it (L.V.); l.salerno@unict.it (L.S.)

[2] Department of Biomedical and Biotechnological Sciences, Section of Pharmacology, School of Medicine, University of Catania, Via Santa Sofia 97, 95123, Catania, Italy; chiara.platania@unict.it (CBMP); claudio.bucolo@unict.it (C.B.)

[3] Department of Molecular Medicine, College of Medicine and Medical Sciences, Princess Al-Jawhara Centre for Molecular Medicine, Arabian Gulf University, Manama 329, Bahrain; khaledfg@agu.edu.bh (K.G.)

[*] Correspondence: vpittala@unict.it; Tel.: +39-095-738-4269

Received: date; Accepted: date; Published: date

Table of contents

^{1}H NMR of compound **1b** (Fig. S1). S2

^{13}C NMR of compound **1b** (Fig. S2). S2

^{1}H NMR of compound **1c** (Fig. S3). S3

^{13}C NMR of compound **1c** (Fig. S4). S3

^{1}H NMR of compound **1m** (Fig. S5). S4

^{13}C NMR of compound **1m** (Fig. S6). S4

^{1}H NMR of compound **1n** (Fig. S5). S5

^{13}C NMR of compound **1n** (Fig. S5). S5

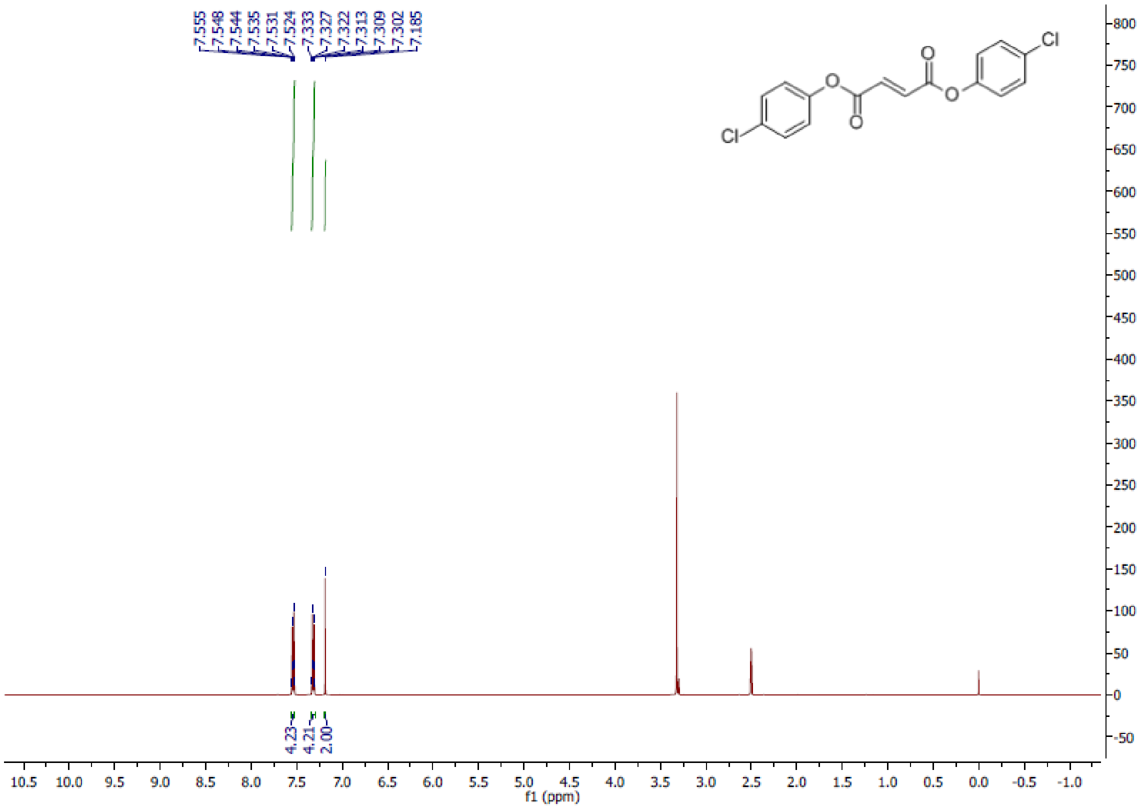

Figure S1. ^{1}H NMR of compound **1b**.

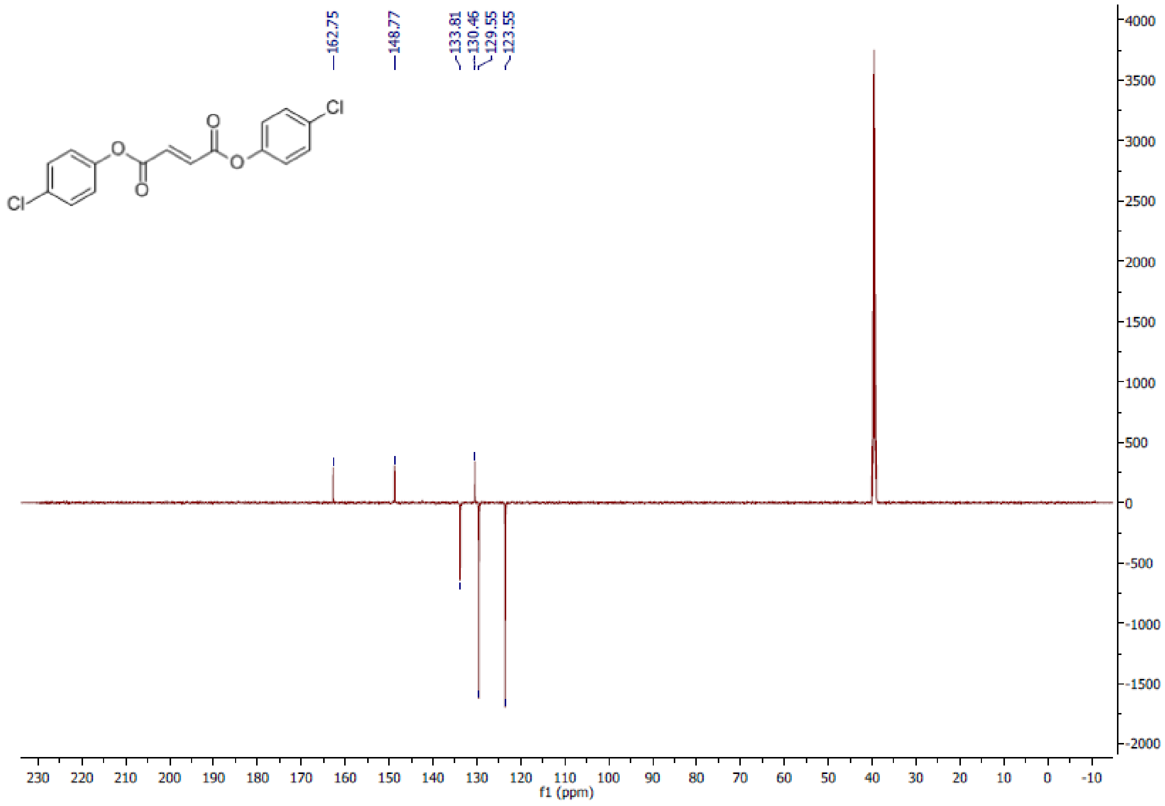

Figure S2. ^{13}C NMR of compound **1b**.

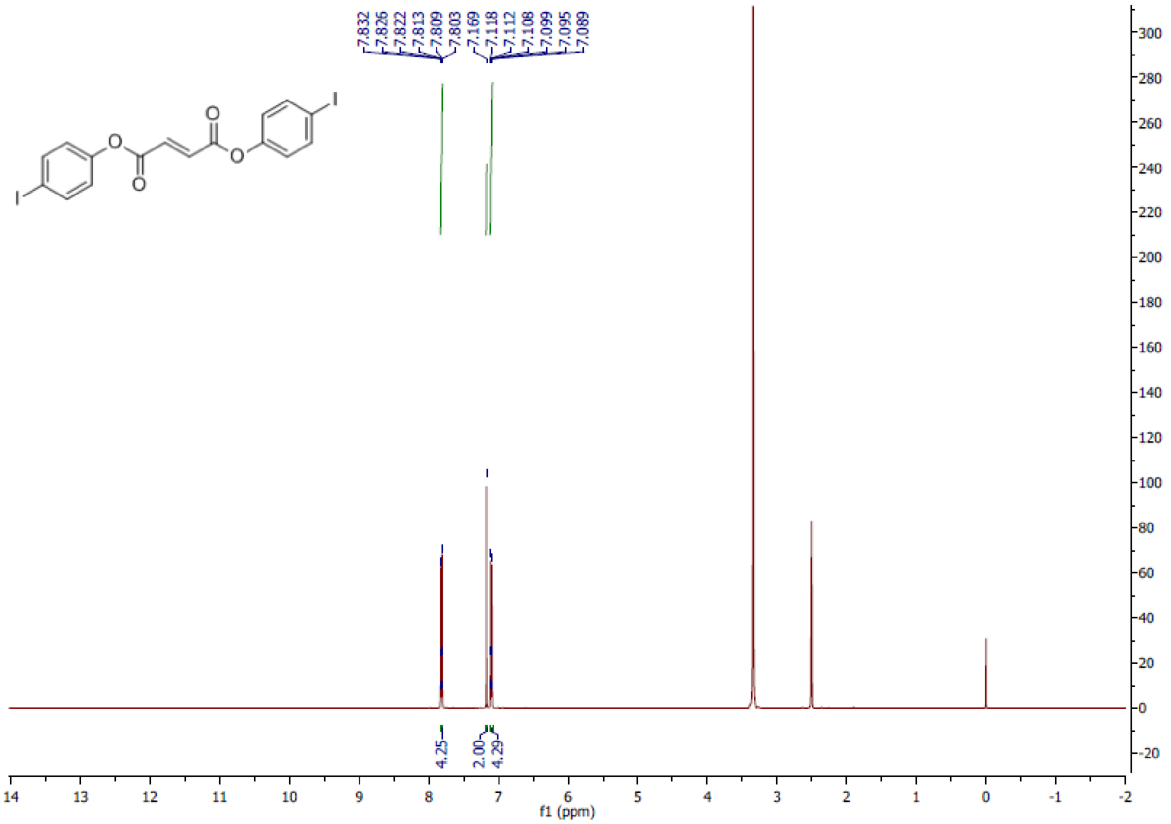

Figure S3. ^{1}H NMR of compound **1c**.

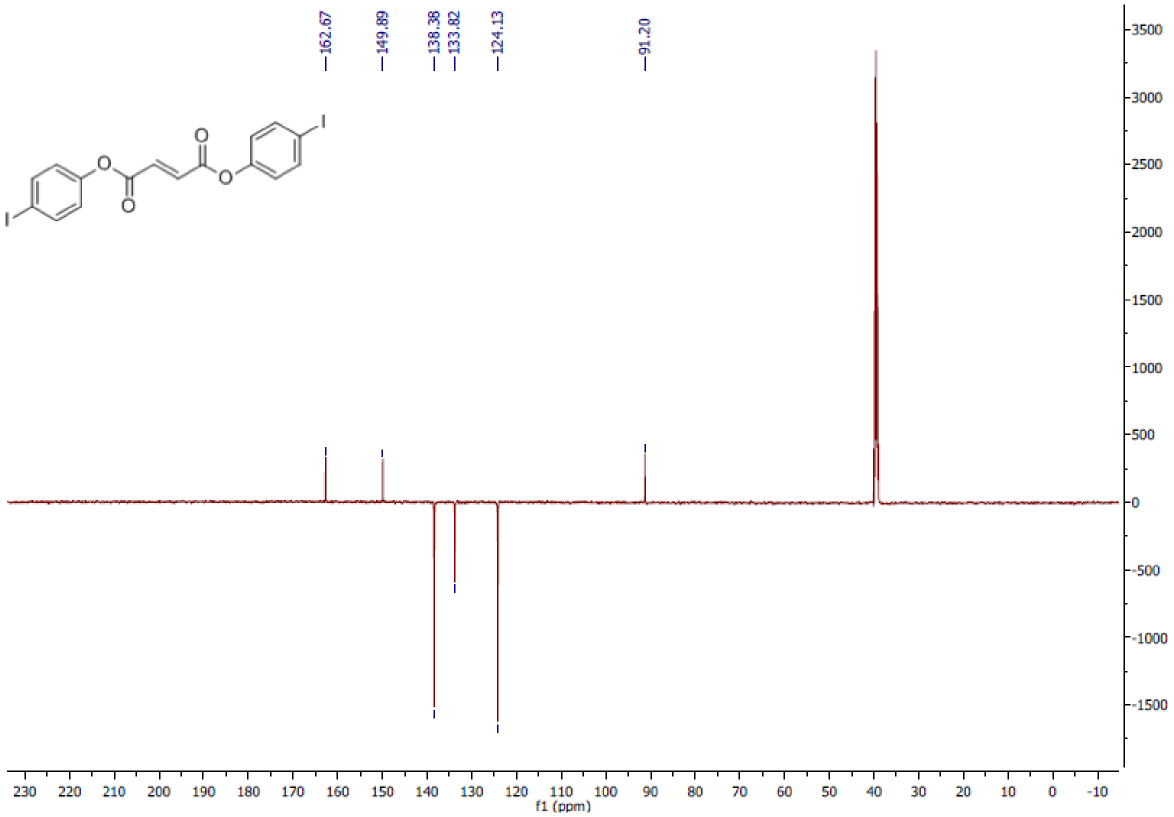

Figure S4. ^{13}C NMR of compound **1c**.

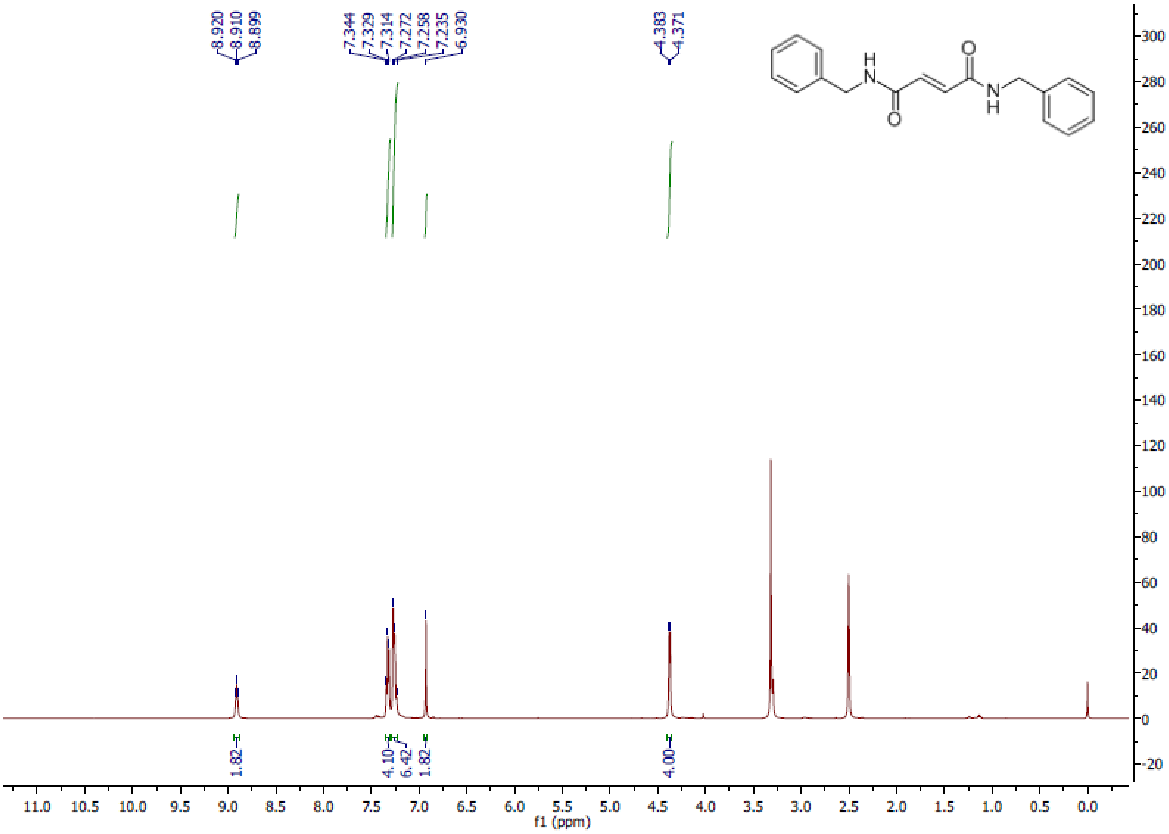

Figure S5. ^{1}H NMR of compound **1m**.

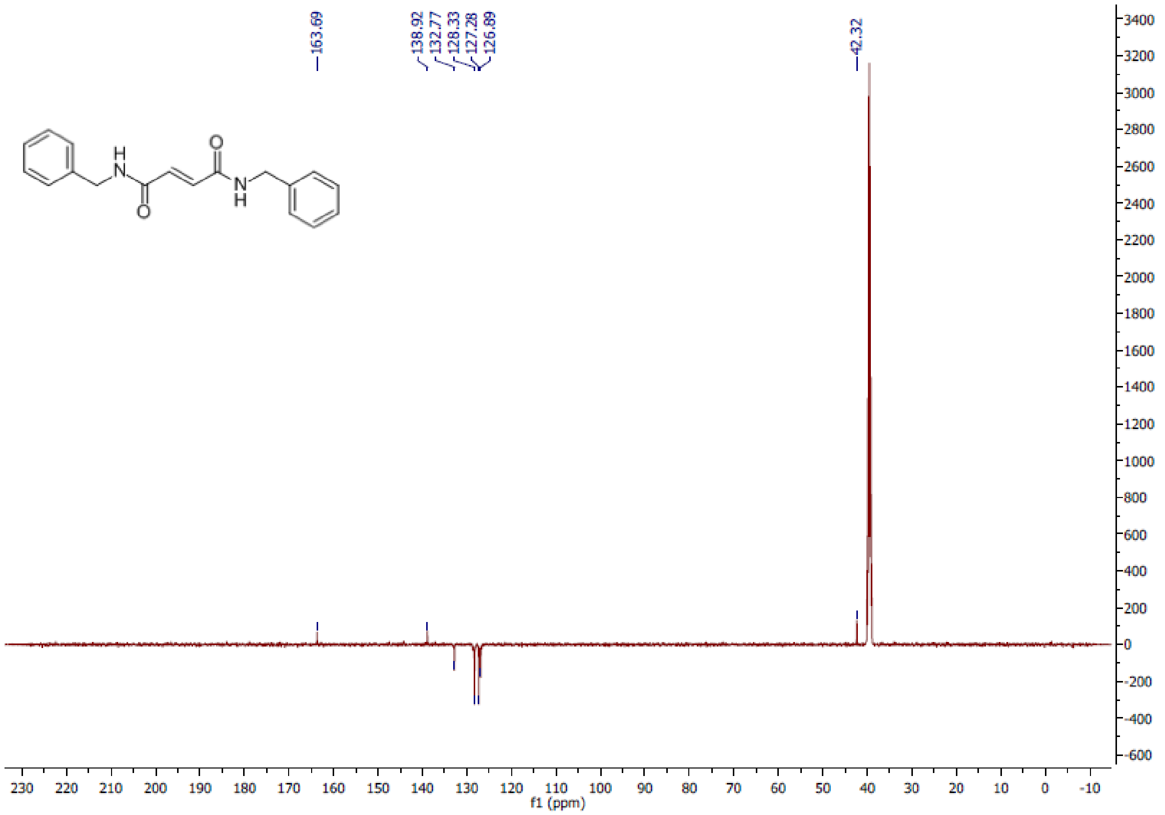

Figure S6. ^{13}C NMR of compound **1m**.

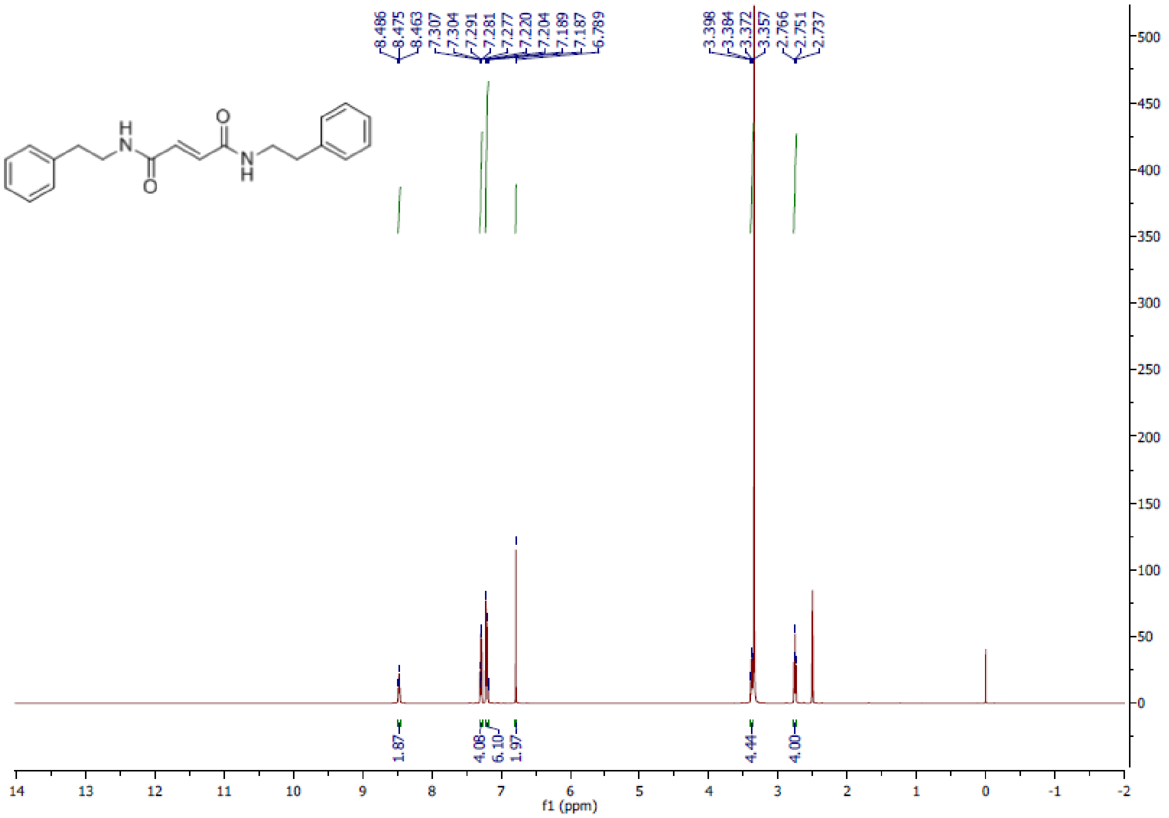

Figure S7. ^{1}H NMR of compound **1n**.

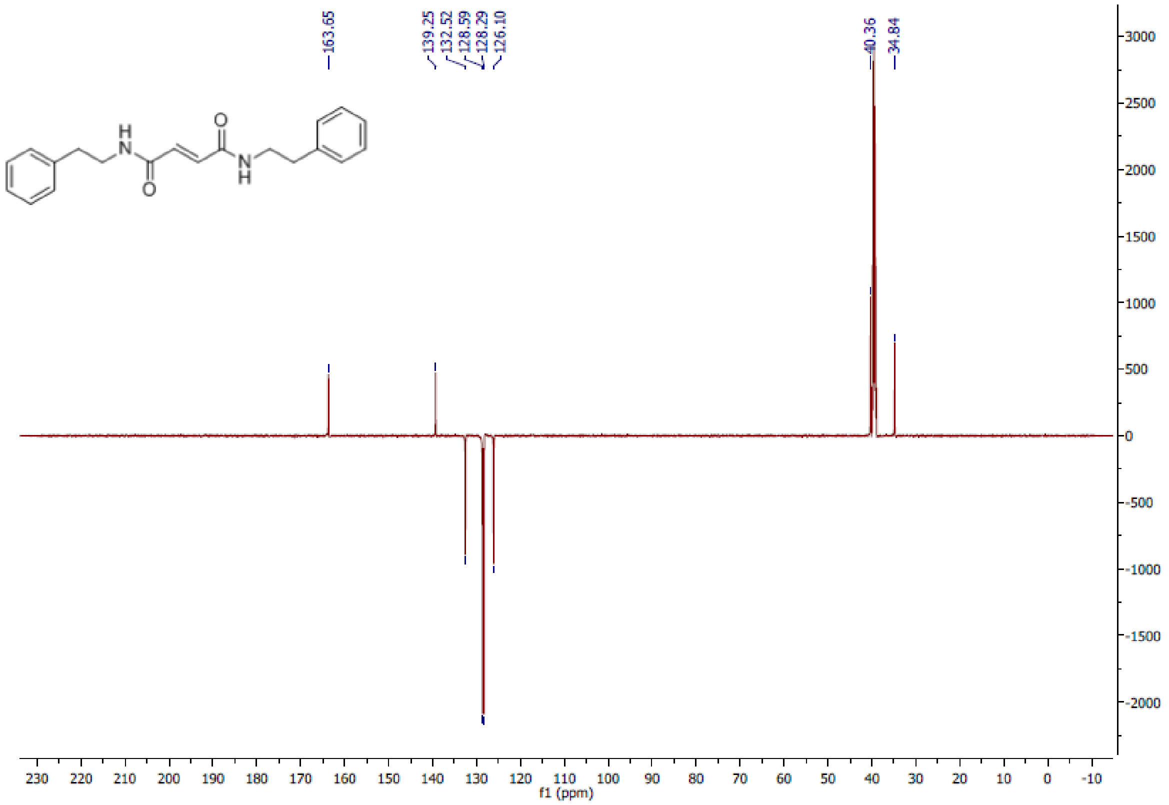

Figure S8. ^{13}C NMR of compound **1n**.
